# Supplementary material for: Bubble explosion induced melt pool instabilities in electron beam melting of aluminum alloy
Source: Nat Commun. 2026 Apr 7;17:4944. doi: 10.1038/s41467-026-71118-3 (PMC13233914; doi:10.1038/s41467-026-71118-3)
Supplement: Supplementary file 1 — Supplementary Information [file 41467_2026_71118_MOESM1_ESM.pdf]

Supplementary Information

Bubble explosion induced melt pool instabilities in electron beam melting of aluminum alloy

Jiandong Yuan [a, b], Luis I. Escano [a, b], Samuel J. Clark [c], Junye Huang [a, b], Ali Nabaa [a, b], Qingyuan Li [a, b], Kamel Fezzaa [c], Lianyi Chen [a, b *]

[a] Department of Mechanical Engineering, University of Wisconsin-Madison, Madison, WI 53706, USA

[b] Department of Materials Science and Engineering, University of Wisconsin-Madison, Madison, WI 53706, USA

[c] X-ray Science Division, Advanced Photon Source, Argonne National Laboratory, Lemont, IL 60439, USA

* Corresponding author

E-mail address: lianyi.chen@wisc.edu

Supplementary Note 1: Dynamics of keyhole fluctuation

One representative keyhole fluctuation cycle is shown in Supplementary Fig. 1. At t_0 + 20 μs, the keyhole opening contracts, reducing keyhole internal energy deposition. This leads to a collapse in the lower region of the keyhole and the formation of a keyhole pore. At t_0 + 40 μs, the contracted keyhole opening is directly irradiated by the electron beam, generating a subsurface bubble. Meanwhile, the internal pressure remains insufficient, causing further collapse and the formation of a second keyhole pore at half depth. At t_0 + 60 μs, the electron beam re-drills the keyhole, allowing it to capture the upper pore. At t_0 + 80 μs, the keyhole continues to grow downward, allowing it to capture the pore below.

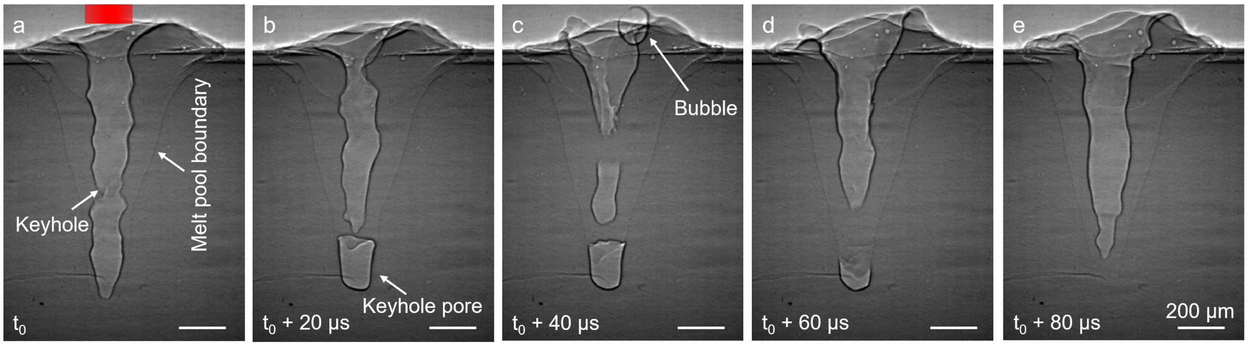

Supplementary Figure 1. Dynamics of periodic keyhole fluctuation during electron beam stationary melting with an electron beam power of 426 W. (a-c) X-ray images showing keyhole collapse and keyhole pore formation during electron beam stationary melting. **(d, e)** X-ray images showing keyhole redrilling and pore elimination during electron beam stationary melting.

Supplementary Note 2: Dynamics of spattering

In addition to the spattering behavior associated with bubble formation and explosion shown in Fig. 6 of the main text, a similar sequence is presented in Supplementary Fig. 2 and Supplementary Movie 8. Immediately after a subsurface bubble explosion (Supplementary Fig. 2a and b), the melt pool becomes unstable within the subsequent frames, and a liquid ligament is ejected from the explosion site (Supplementary Fig. 2c and d), leading to spatter formation (Supplementary Fig. 2e and f).

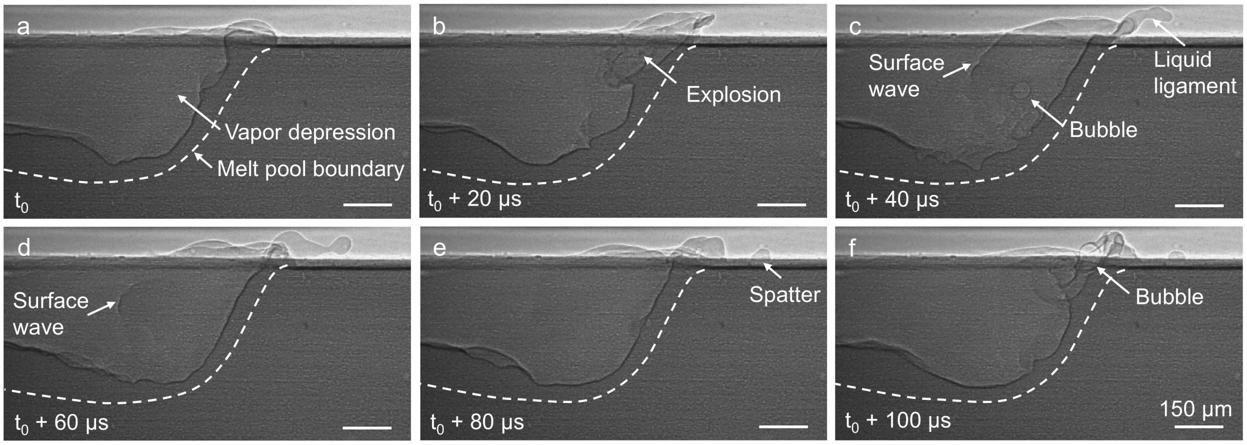

Supplementary Figure 2. Dynamics of spattering under the influence of subsurface explosion during electron beam scanning with an electron beam power of 426 W and a scan speed of 1.0 m/s. (a, b) X-ray images showing bubble explosions on front vapor depression wall during electron beam scanning. **(c-f)** X-ray images showing the dynamics of liquid ligament and spattering induced by bubble explosions during electron beam scanning. Prior to melting, a preheating step was performed using the electron beam operated in pulse mode with a 0.4 duty cycle, a power of 318 W, a scan speed of 16 m/s, and a duration of 10 s. The duty cycle of a pulsed electron beam is the fraction of each pulse period during which the beam is actively on, defined as: $Duty\ cycle = \frac{t_{on}}{t_{on}+ t_{off}}$, where t_{on} and t_{off} are the durations of the beam-on and beam-off periods within one pulse, respectively.

Supplementary Note 3: Influence of preheating on melting dynamics

The influence of preheating on melting dynamics, including subsurface bubble formation, melt pool behavior, and vapor depression evolution was investigated. Preheating was carried out by electron beam scanning with a lower electron beam power and a fast speed before melting. Subsurface bubble formation and explosions were observed, accompanied by melt pool dynamics similar to those observed without preheating (Supplementary Fig. 3a). Driven by the Plateau-Rayleigh and explosion-induced instabilities, this elongated melt pool eventually forms a humping structure on the melt track after solidification (Supplementary Fig. 3b-d).

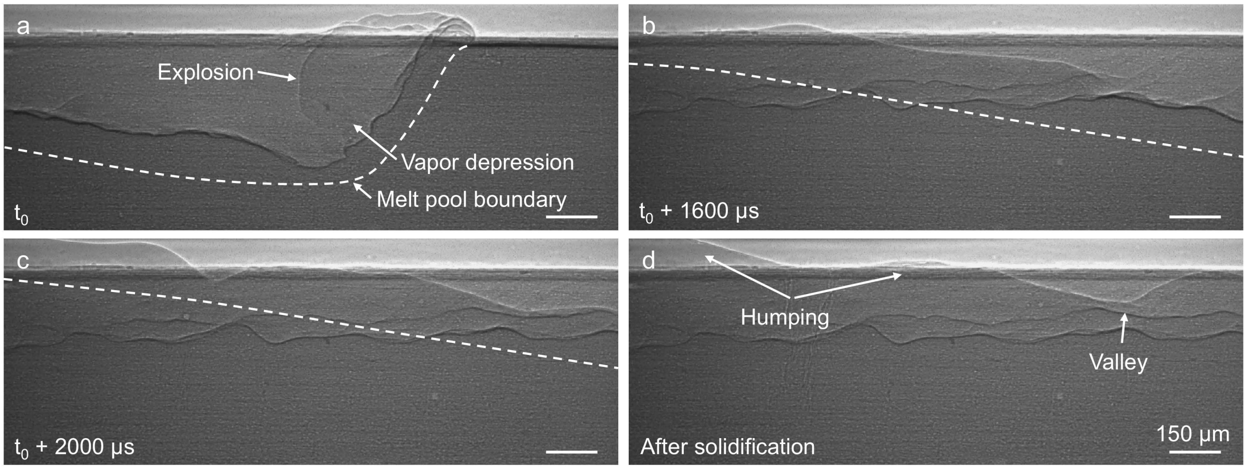

Supplementary Figure 3. Melting dynamics during electron beam scanning with preheating. (a-c) X-ray images showing the bubble explosion and melt pool dynamics during electron beam scanning. **d** X-ray image showing periodic humping after solidification. Prior to melting, a preheating step was performed using the electron beam operated in pulse mode with a 0.4 duty cycle, a power of 318 W, a scan speed of 16 m/s, and a duration of 10 s. Melting was then performed with an electron beam power of 426 W and a scan speed of 1.0 m/s.

Supplementary Note 4: Image processing

The method of image processing is illustrated in Supplementary Fig. 4. To enhance contrast, the image intensity at each pixel of Frame (i + 1) was divided by the intensity of corresponding pixel in Frame (i) (Supplementary Fig. 4a, b), effectively removing static regions and rendering them as a blank background (Supplementary Fig. 4c). After dividing, the bubbles from Frame (i + 1) appear bright in the processed image, while those from Frame (i) appear dark, making their movement more distinct. Additionally, the melt pool boundary and surface waves at the vapor–liquid interface become more pronounced.

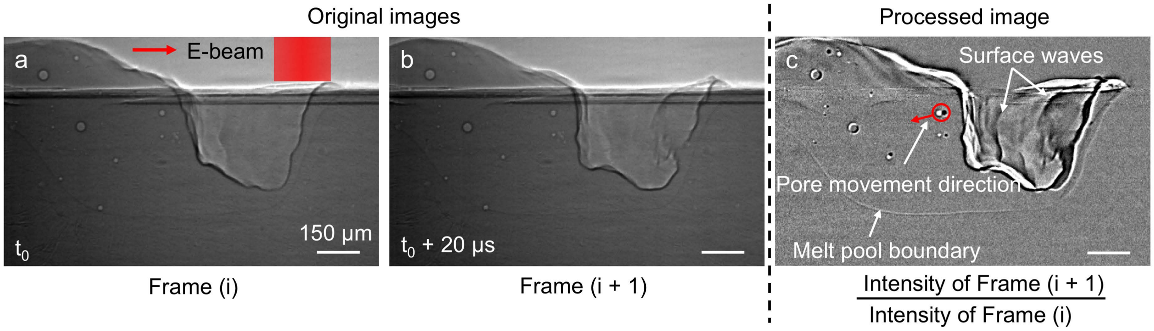

Supplementary Figure 4. Image processing method. **(a, b)** Original X-ray images obtained at Frame (i) and Frame (i + 1). **c** Processed X-ray image to show surface waves and pore movement trajectory. The image intensity at each pixel of Frame (i + 1) is divided by the intensity of corresponding pixel in Frame (i).
