# Supplementary material for: Bubble explosion induced melt pool instabilities in electron beam melting of aluminum alloy
Source: Nat Commun. 2026 Apr 7;17:4944. doi: 10.1038/s41467-026-71118-3 (PMC13233914; doi:10.1038/s41467-026-71118-3)
Supplement: Supplementary file 2 — Description of Additional Supplementary Files [file 41467_2026_71118_MOESM2_ESM.pdf]

Description of Additional Supplementary Files

File Name: Supplementary Movie 1
Description: Dynamics of subsurface bubble formation and explosion during electron beam stationary melting with an electron beam power of 109 W.

File Name: Supplementary Movie 2
Description: Dynamics of subsurface bubble formation and explosion during electron beam scanning under a shallow vapor depression at 318 W electron beam power and 1 m/s scan speed.

File Name: Supplementary Movie 3
Description: Dynamics of subsurface bubble formation and explosion during electron beam scanning under a deep depression at 426 W electron beam power and 0.7 m/s scan speed.

File Name: Supplementary Movie 4
Description: Initiation of keyhole and melt pool under the influence of subsurface explosion during electron beam stationery melting with an electron beam power of 426 W.

File Name: Supplementary Movie 5
Description: Evolution of the vapor depression and melt pool under the influence of subsurface explosion during electron beam scanning with an electron beam power of 426 W and a high scan speed of 1.2 m/s.

File Name: Supplementary Movie 6
Description: Evolution of the vapor depression and melt pool under the influence of subsurface explosion during electron beam scanning with an electron beam power of 426 W and a low scan speed of 0.6 m/s.

File Name: Supplementary Movie 7
Description: Dynamics of spattering under the influence of subsurface explosion during electron beam scanning with an electron beam power of 385 W and a scan speed of 1.0 m/s.

File Name: Supplementary Movie 8
Description: Dynamics of spattering under the influence of subsurface explosion during electron beam scanning with an electron beam power of 426 W and a scan speed of 1.0 m/s. Prior to melting, a preheating step was performed using the electron beam operated in pulse mode with a 0.4 duty cycle, a power of 318 W, a scan speed of 16 m/s, and a duration of 10 s.

File Name: Supplementary Movie 9
Description: Dynamics of subsurface bubble formation and explosion on the front keyhole wall during electron beam scanning with an electron beam power of 318 W and a scan speed of 0.3 m/s.

File Name: Supplementary Movie 10

Description: Dynamics of bubble formation, explosion and avalanche during electron beam scanning with an electron beam power of 318 W and a scan speed of 0.7 m/s.
